# Electrospun Fe_3_O_4_-Sn@Carbon Nanofibers Composite as Efficient Anode Material for Li-Ion Batteries

**DOI:** 10.3390/nano11092203

**Published:** 2021-08-27

**Authors:** Hong Wang, Yuejin Ma, Wenming Zhang

**Affiliations:** 1 College of Mechanical and Electrical Engineering, Hebei Agricultural University, Baoding 071001, China; wanghongmail@sina.com; 2 College of Electronic Information Engineering, Hebei University, Baoding 071002, China; 3 National-Local Joint Engineering Laboratory of New Energy Photoelectric Devices, College of Physics Science and Technology, Hebei University, Baoding 071002, China

**Keywords:** Fe_3_O_4_, Sn, carbon nanofibers, lithium-ion batteries, anode material

## Abstract

Nanoscale Fe_3_O_4_-Sn@CNFs was prepared by loading Fe_3_O_4_ and Sn nanoparticles onto CNFs synthesized via electrostatic spinning and subsequent thermal treatment by solvothermal reaction, and were used as anode materials for lithium-ion batteries. The prepared anode delivers an excellent reversible specific capacity of 1120 mAh·g^−1^ at a current density of 100 mA·g^−1^ at the 50th cycle. The recovery rate of the specific capacity (99%) proves the better cycle stability. Fe_3_O_4_ nanoparticles are uniformly dispersed on the surface of nanofibers with high density, effectively increasing the electrochemical reaction sites, and improving the electrochemical performance of the active material. The rate and cycling performance of the fabricated electrodes were significantly improved because of Sn and Fe_3_O_4_ loading on CNFs with high electrical conductivity and elasticity.

## 1. Introduction

With the development of the social economy, the imbalance of energy supply and demand has become prominent. The development of new energy and new energy-materials has become an important topic. Lithium-ion batteries, as electrochemical power sources, have been widely used in mobile phones, laptops, cameras, and other portable electronic devices [1,2,3], hybrid electric vehicles (HEVs), and plug-in hybrid electric vehicles (PHEVs), due to their advantages of environmentally friendliness, high capacity, high power density, high safety, long cycle life, and so on. However, graphite, the most common commercial anode material, only has a theoretical lithium storage specific capacity of 372 mAh·g^−1^ [4,5,6], which is far below the application requirements. Therefore, finding alternative anode materials with better electrochemical performance, such as silicon-based composites [7,8,9], metal intermetallic alloys [10,11,12,13,14], and transition metal oxides [15,16,17], has become increasingly important [18,19,20]. Among these anode materials, cobaltous oxide and tin are very promising candidates to replace graphite, and have attracted the attention of many scholars. Fe_3_O_4_, as a transition metal oxide (M_x_O_y_, M = Co, Cu, Fe, Mn), is well-known because of its high theoretical lithium storage capacity (890 mAh·g^−1^), great safety performance, nontoxicity, and low cost [21]. In addition, Sn, as a metal, also has a high theoretical specific capacity (994 mAh·g^−1^) [22,23]. However, the practical application of Fe_3_O_4_ and Sn still faces a series of enormous problems, namely, rapid capacity decay and poor capacity retention ability and cycle performance, owing to the large volume changes of the material occurring during the lithium insertion/de-insertion process. Fe_3_O_4_, for example, will generate approximately 200% of the volume change, and lead to pulverization due to the action of internal stress. In addition, the volume expansion also causes an increase in electrical contact resistance and results in a decrease in the electrochemical performance of the material [24,25,26,27,28].

Studies on Fe_3_O_4_ and Sn anodes of lithium-ion batteries have mainly focused on improving the cycle life and reversible capacity by nanometer materials and compounding with other elastic materials. Optimization of the nanostructure and preparation of composite materials can effectively inhibit volume expansion, reduce the electrical contact resistance, and improve the electrochemical performance of materials. Associated preparation methods of synthesizing revolutionary nanocomposites include electrospinning [29], pyrolysis [30], templating [31], chemical vapor deposition [32], sol-gel methods [33], and so on. A series of Fe_3_O_4_-based nanomaterials were prepared, such as Fe_3_O_4_ nanowire arrays [34], Fe_3_O_4_/graphene composites [35,36], Fe_3_O_4_/carbon nanotube composites [37], hexahedral Fe_3_O_4_ [38], and hollow Fe_3_O_4_ spheres. [39]. The same strategies can also be applied to the optimization of Sn, such as improving the discharge capacity through the preparation of Sn-M-C composites (M = Fe, Ni, CO) [40,41,42,43] to inhibit volume expansion via N-doped Sn@carbon composites [44], tin alloys [45,46,47], and tin balls. [48,49]. Carbon nanofibers (CNFs) with high electrical conductivity and surface area are suitable for preparing composites, and have been used in many areas, such as sodium-ion batteries [50], direct methanol fuel cells [51], selective detection of dopamine [52], biosensors [53]. The excellent microstructure of the CNFs which have higher conductivity, and a larger specific surface area to complete better electrical contact between the electrode and electrolyte, and have good structural stability to provide more space for the expansion and contraction of materials, is beneficial to improve the electrochemical performance. All of these factors will contribute to improving the performance of lithium-ion batteries.

Therefore, a novel composite comprising Fe_3_O_4_-Sn@CNFs was fist synthesized by electrostatic spinning and solvothermal reaction. When utilized as anode electrode material for lithium-ion batteries, this novel Fe_3_O_4_-Sn@CNFs electrode exhibits an excellent reversible specific capacity of 1120 mAh·g^−1^ at a current density of 100 mA g^−1^. The recovery rate of the specific capacity (99%) proves the better cycle stability. This work plays a guiding role in the development of lithium-ion batteries.

## 2. Experiment Procedure

### 2.1. Preparation of Carbon Nanofibers

In a typical preparation, 1 g polyacrylonitrile (PAN, M_w_ = 150,000, Sigma-Aldrich Co., Shanghai, China) was dissolved in 9 g N, N-dimethylformamide (DMF) (Aladdin Co., Shanghai, China) and then stirred to form a homogeneous transparent polymer solution at room temperature. The result solution was then transferred to five 10-mL syringes with needles connected to the anode of a high-voltage DC power supply. Electrospun nanofibers were collected on a roller wrapped with aluminum foil, and the distance between the tip and the collector was 15 cm. The applied voltage was 27 kV, and the flow rate of the solution was 15 μL min^−1^. The obtained fibers were first heated to 270 °C at a rate of 1 °C min^−1^ in air to stabilize the fibers, and kept at 270 °C for 1 h. Then they were carbonized by continued heating to 1000 °C at a rate of 5 °C min^−1^ in nitrogen atmosphere and kept for 1 h. The obtained products were denoted as CNFs.

### 2.2. Preparation of Sn@CNFs

The preparation approaches of Sn loading on carbon nanofibers are similar to those of CNFs except that: tin (II) acetate is first added to the prepared PAN and DMF mixed solution and then stirred for 12 h to obtain the homogeneous solution. Second, in the heat treatment, stabilization was carried out at 230 °C for 1 h at a rate of 1 °C min^−1^, and carbonization was carried out at 800 °C for 1 h. The obtained products were denoted as Sn@CNFs.

### 2.3. Preparation of Fe_3_O_4_@CNFs

The solvothermal route used in this work has been reported in previous work [54,55]. In a typical synthesis, 40 mg CNFs was dispersed in 20 mL DMF under sonication for 2 h to form a uniform black solution at a concentration of 2 mg mL^−1^. Then, 10 mL tetraethylene glycol (TEG) (Aladdin Co., Shanghai, China) and 100 mg iron (III) acetylacetonate (Fe(acac)_3_) (Aladdin Co., Shanghai, China) were added to the solution, and stirred for 30 min. The mixed solution was transferred to a Teflon-lined stainless steel autoclave (Yipin Technology Co., Shanghai, China) to proceed with solvothermal reaction at 180 °C for 2 h. After the solvothermal reaction was completed, the autoclave was cooled to room temperature. The product of Fe_3_O_4_@CNFs was collected after centrifugation, washed with deionized water three times, and finally dried in a vacuum oven at 60 °C overnight.

### 2.4. Preparation of Fe_3_O_4_-Sn@CNFs

The preparation method was the same as Section 2.3, except the CNFs were replaced with Sn@CNFs.

### 2.5. Preparation of Fe_3_O_4_ Powder

The same method was used to prepare Fe_3_O_4_ powder as described in Section 2.3, just without adding the CNFs powder.

### 2.6. Characterization of Materials

The surface morphology of all samples was examined by scanning electron microscopy (SEM, NOVA NANOSEM450, FEI Co., Ltd., Hillsborough, NC, USA). The size, distribution, and coating of the nanoparticles were observed by transmission electron microscopy (TEM, JEM-2000 FXII, JEOL Ltd., Tokyo, Japan). X-ray diffraction (XRD, D8 ADVANCE, Bruker AXS Co., Karlsruhe, Germany) analysis of samples for the chemical composition and the degree of crystallinity were performed with Cu Kα radiation (λ = 0.154 nm). The elemental distribution of Fe_3_O_4_-Sn@CNFs was examined by X-ray photoelectron spectroscopy (XPS, ESCALAB 250Xi, Thermo Fisher Scientific Inc., Waltham, MA, USA). The electrochemical performances were determined using a coin cell (type CR2032), which was assembled in a glove box filled with argon. Copper foil was coated with a slurry of 80 wt% active material, 10 wt% super P (as the conductive agent), and 10 wt% hydroxymethyl cellulose (CMC, as the binder) dissolved in a proper amount of NMP to form the working electrode. Lithium foil was used as the counter and reference electrodes and a microporous polymer separator (Celgard^®^ 2400) (Celgard LLC, Charlotte, NC, USA) was placed between the two electrodes. The electrolyte was a solution of 1 M LiPF_6_ dissolved in a mixed solution of ethylenecarbonate (EC) and dimethyl carbonate (DMC) (1:1 by volume) (Zhangjiagang Guotai Huarong Chemical New Material Co., Zhanjiagang, China). The battery case and polypropylene film must be dried at 100 °C in a vacuum to remove oxygen and water, and the amount of oxygen and water in the glove box must be less than 0.1 ppm. Electrochemical impedance spectroscopy (EIS) measurements were used to test the impedance changes, and cyclic voltammetry (CV) measurements were used to study the process of the electrochemical reactions, both of which were conducted on a CHI660E electrochemical workstation (CH Instruments, Shanghai, China). The discharge and charge test, including cycling and the rate performance, was conducted on the LAND-CT2001A battery cycle system (LAND Electronics, Wuhan, China).

## 3. Results and Discussion

**Figure 1:**
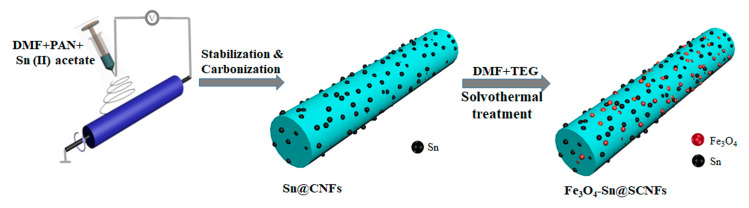
Schematic illustration of the preparation process for Fe_3_O_4_@Sn-CNFs.

The novel Fe_3_O_4_-Sn@CNFs composite nanomaterial was fabricated via a simple electrospinning and subsequent calcination process combined with a hydrothermal method. Figure 1 shows a schematic illustration of the preparation of Fe_3_O_4_-Sn@CNFs. Electrospinning is a convenient and important technique to prepare continuous one-dimensional carbon nanofibers [56,57,58,59] that were used in this study to synthesize Sn loading on carbon nanofibers (Sn@CNFs), which were then coated with Fe_3_O_4_ by a solvothermal method. Fe_3_O_4_ is distributed uniformly at a high density on the surface of fibers with nanoscale diameters. CNFs, as elastic materials characterized by flexibility and ductility, can alleviate the volume changes of Fe_3_O_4_ and Sn during the lithium insertion/de-insertion process, and maintain structural stability. Concurrently, the presence of Sn@CNFs enhanced the electrical conductivity of the material and promoted the transfer of electrons and ions. More importantly, the high density of nanoscale Fe_3_O_4_ particles and Sn provided more active sites, which greatly improved the specific capacity of the composite. Therefore, the as-prepared Fe_3_O_4_-Sn@CNFs nanocomposites exhibited high reversible specific capacity, better rate capability, and excellent cycling durability.

**Figure 2:**
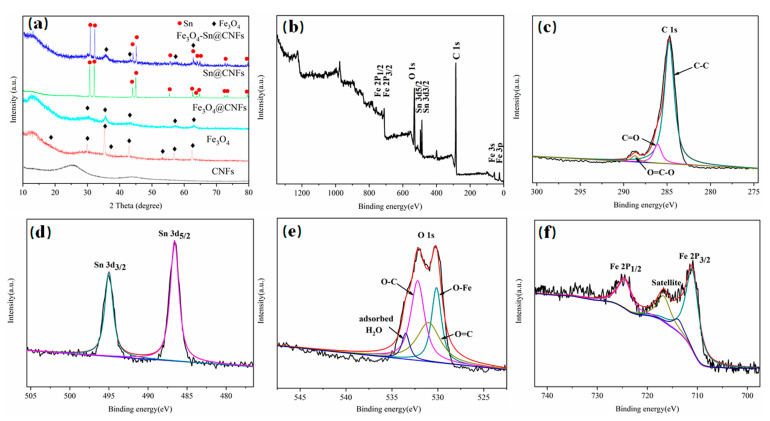
(**a**) The crystalline texture of CNFs, Sn@CNFs, Fe_3_O_4_, Fe_3_O_4_@CNFs, and Fe_3_O_4_-Sn@CNFs analyzed by X-ray diffraction (XRD). (**b**–**f**) The XPS pattern of Fe_3_O_4_@Sn-CNFs composite nanofibers and the different elements in different electron orbitals.

As shown in Figure 2a, the crystalline textures of CNFs, Sn@CNFs, Fe_3_O_4_, Fe_3_O_4_@CNFs, and Fe_3_O_4_-Sn@CNFs were analyzed by X-ray diffraction (XRD). The XRD pattern of the CNFs reveals two broad and smooth peaks approximately at 2θ = 25° and 44°; the former peak proved the formation of typical amorphous carbon [60,61], and the latter weaker peak indicated the presence of a little ordered carbon in the CNFs [62]. The XRD pattern of the Fe_3_O_4_ powder exhibited diffraction peaks at 2θ = 18.2°, 30.0°, 35.4°, 37.0°, 43.0°, 53.4°, 56.9°, and 62.5°, corresponding to the (111), (220), (311), (222), (400), (422), (511), and (440) planes, respectively, indicating a cubic Fe_3_O_4_ structure with a space group of F*d*-3*m* (JCPDS No. 89-0688). In addition, compared to Fe_3_O_4_ powder, the Fe_3_O_4_ peaks of Fe_3_O_4_@CNFs and Fe_3_O_4_-Sn@CNFs were weaker in intensity, indicating that Fe_3_O_4_ did not accumulate on the surface of the fibers, but formed a small nanoscale structure. The corresponding peaks at 2θ = 30.6°, 32.0°, 43.9°, 44.9°, 55.3°, 62.5°, 63.8°, 64.6°, 72.4°, 73.1°, and 79.5° could be indexed to the (200), (101), (220), (211), (301), (112), (400), (321), (420), (411), and (312) planes of Sn metal, respectively, and were accordant with a tetragonal structure, expressing a space group of I4_1_/amd (JCPDS No. 65-0296). Fe_3_O_4_-Sn@CNFs have a similar peak of Sn as Sn@CNFs, implying the microstructure of Sn has not been influenced by the solvothermal reaction and subsequent treatment process. The decrease in peak intensity and SEM results proved that many Fe_3_O_4_ particles were coated on the Sn@CNFs and constituted a Fe_3_O_4_ shell.

The XPS diagram of Fe_3_O_4_-Sn@CNFs is shown in Figure 2b, which revealed the presence of Fe, C, O, and Sn elements. All binding energies were calibrated by referencing the C 1s peak at 284.8 eV. The C 1s spectrum (Figure 2c) can be deconvoluted into three peaks, located at 288.7 eV, 286.1 eV, and 284.8 eV, which correspond to the chemical bonds (O=C–O and O=C) formed by O elements remaining in the preparation and calcination process and the C–C chemical bond in CNFs themselves [63,64]. The peaks at 495 eV and 486.5 eV in Figure 2d correspond to Sn 3d3/2 and Sn 3d5/2 respectively, which proved the presence of Sn in the Fe_3_O_4_-Sn@CNFs samples [65]. Figure 2e shows the oxygen element in the XPS pattern of the O 1s spectrum diagram, and two peaks can be deconvoluted into four peaks. The peak at 530.2 eV corresponds to the Fe-O bond in the crystalline phase of Fe_3_O_4,_ and the different combinations of O and C states (O=C and O–C chemical bonds) correspond to two neighboring peaks at 531 eV and 532.2 eV respectively. The higher peak at 533.5 eV may be attributed to the O–H bond of the adsorbed water molecules [66]. Figure 2f shows the Fe 2p spectrum of two peaks located at 725 eV and 710.3 eV, corresponding to Fe 2p1/2 and Fe 2p3/2 respectively, with a satellite peak at 717.5 eV, confirming the presence of Fe [67].

**Figure 3:**
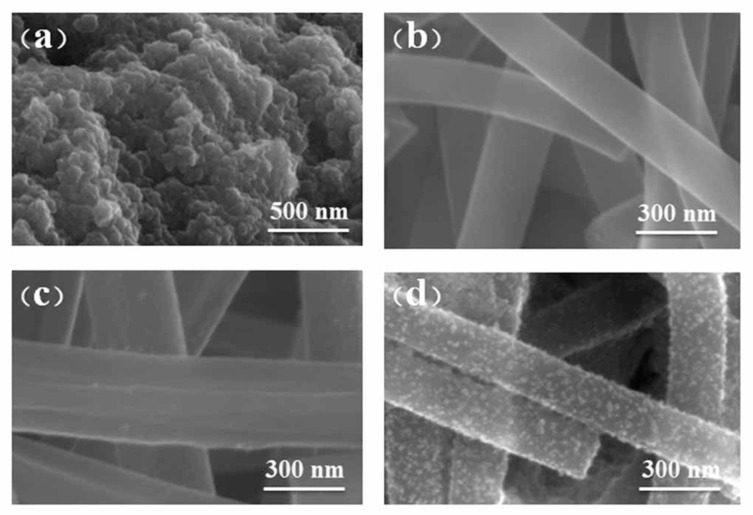
The SEM images of the surface topography of (**a**) Fe_3_O_4_ powder at 500 nm and (**b**) CNFs, (**c**) Sn@CNFs, (**d**) Fe_3_O_4_-Sn@CNFs at 300 nm.

Figure 3 shows the SEM images of the synthesized Fe_3_O_4_ powder, CNFs, Sn@CNFs, and Fe_3_O_4_-Sn@CNFs products. As shown in Figure 3a, Fe_3_O_4_ nanoparticles with a diameter of several nanometers agglomerated together to generate larger particles (with an average diameter approximately 50 nm) because of magnetism. CNFs (Figure 3b) and Sn@CNFs (Figure 3d) have interconnecting and overlapping morphologies and uniform diameters (approximately 260 nm), which enhance the electrical conductivity to a certain extent. The CNFs in Figure 3b can be clearly observed to have a smooth surface, indicating that PAN (as the precursor of carbon) was blended well with DMF (as solvent) and turned into fibers after the electrospinning and calcining process. The surface of Sn@CNFs (Figure 3c) was rugged because of the tin(II) acetate transforming to metal Sn after the calcining process and some of the Sn nanoparticles were exposed in the air which destroyed the surface of nanofiber. The morphology of Fe_3_O_4_-Sn@CNFs is shown in Figure 3d, Fe_3_O_4_ particles were loaded on the surface of Sn@CNFs at a high density. However, the size increased to approximately 8 nm, which might be due to the effect of internal Sn particles that were exposed to the surface of the fibers on the solvothermal reaction. The increase in density and particle size combined with the increase in the content of Fe_3_O_4_ in the composite enhanced the specific capacity of the active material. All Fe_3_O_4_ nanoparticles grew on the surface of Sn@CNFs, and were barely flocked together or free Fe_3_O_4_ particles, which could improve the use of materials. In addition, the diameter and surface of the CNFs and Sn@CNFs did not change after the solvothermal reaction, indicating that carbon and Sn were not affected by the reaction.

**Figure 4:**
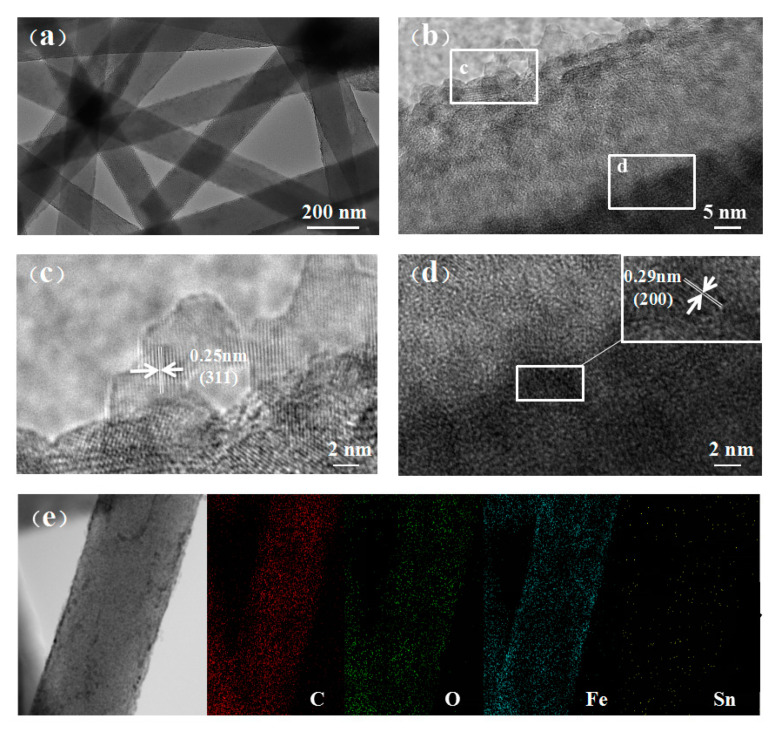
(**a**–**d**) The TEM images and the high-resolution TEM image of Fe_3_O_4_-Sn@CNFs. (**e**) Mapping diagram of elements contains four elements.

TEM and high-resolution TEM (HR-TEM) images of Fe_3_O_4_-Sn@CNFs are shown in Figure 4. The graphic in Figure 4a reveals that the fibers with a diameter of approximately 200 nm cross each other and form a stable network structure. Figure 4b shows the HR-TEM image of the material surface in which the light and dark parts corresponded to the Fe_3_O_4_ shell and Sn@CNFs respectively. The Fe_3_O_4_ shell (Figure 4c) was well crystallized, and the crystalline interplanar spacing of Fe_3_O_4_ nanoparticles was 0.25 nm, which matched well with the lattice spacing of the (311) planes of Fe_3_O_4_. In Figure 4d, the particles that assembled into nanofibers mainly existed in an amorphous state, but some crystalline nanoparticles were still present basically due to the crystallization of Sn. The Sn nanoparticles embedded in the fiber are shown in the upper right corner of the illustrations. The particle diameter was only approximately 1 nm, and the lattice spacing was 0.29 nm, corresponding to the (200) planes of crystalline Sn. No obvious carbon lattice fringes were observed, indicating that mainly amorphous carbon was present. The mapping diagram of the elements in Figure 4e also proves that only a small amount of Sn was exposed at the external surface, and the content of Sn was lower in the material than in the C, O, and Fe elements.

**Figure 5:**
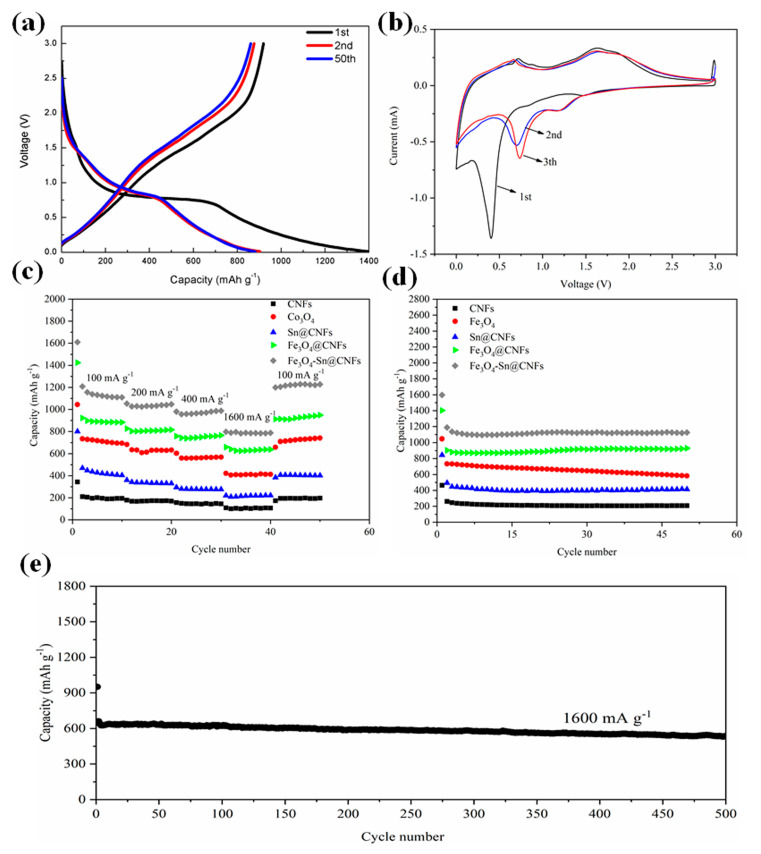
(**a**) Voltage profile of Fe_3_O_4_-Sn@CNFs at 100 mA·g^−1^ in 1 M LiPF_6_/EC/DMC. (**b**) Cyclic voltammetry curves of Fe_3_O_4_-Sn@CNFs at 0.2 mV/s scan rate in 1 M LiPF_6_/EC/DMC. (**c**) Rate performance of CNFs, Sn@CNFs, Fe_3_O_4_, Fe_3_O_4_@CNFs, and Fe_3_O_4_-Sn@CNFs at different current densities. (**d**) Cycle performance and coulombic efficiency of CNFs, Sn@CNFs, Fe_3_O_4_, Fe_3_O_4_@CNFs, and Fe_3_O_4_-Sn@CNFs at 100 mA·g^−1^ in 1 M LiPF_6_/EC/DMC. (**e**) Long-term cycles of Fe_3_O_4_-Sn@CNFs at 1600 mA·g^−1^ current densities.

Figure 5a shows the initial, second, and fiftieth discharge and charge profiles of Fe_3_O_4_-Sn@CNFs composite electrodes with the voltages ranging from 0.01–3 V at a current density of 100 mA·g^−1^. The initial discharge and charge capacity of Fe_3_O_4_-Sn@CNFs were 1597.2 mAh·g^−1^ and 1197 mAh·g^−1^ respectively, with an initial coulombic efficiency of 74.94%, due to the irreversible loss of lithium, and the formation of solid electrolyte interface (SEI) film on the surface of the material during the discharge process. The coulombic efficiency of Fe_3_O_4_-Sn@CNFs steeply increased to 97.4% in the second discharge and charge process, having a discharge capacity of 1187.9 mAh·g^−1^ and charge capacity of 1157 mAh·g^−1^, and then stabilized at a high-coulombic efficiency of approximately 98.5%.

Cyclic voltammetry measurements from 0.01–3 V (vs. Li^+^/Li) of the coin cell fabricated by the Fe_3_O_4_-Sn@CNFs composite anode, performed at a scan rate of 0.2 mV s^−1^, are shown in Figure 5b. During the first cycle, the reduction peak at 0.35 V corresponds to the conversion of Fe_3_O_4_ to Fe and Sn to Li_x_Sn, the formation of amorphous Li_2_O and partially irreversible solid electrolyte interface (SEI) film on the surface of the materials. In addition, two weak peaks observed at approximately 0.7 V and 1.4 V can be assigned to the formation of Li_x_Fe_3_O_4_ [68,69]. The oxidation peak observed at 0.7 V was generated from the appearance of Sn during the Li^+^ deintercalation process, and the broad peak at 1.5–1.9 V was related to the reduction of Fe^3+^/Fe^2+^ to Fe as described in Equations (1) and (2), respectively. In the second cycle, the relatively weak peak observed at 0.75 V, which was related to the conversion of Fe_3_O_4_ to Fe and Li_x_Sn to Sn, replaced the sharp peak at 0.35 V, indicating the formation of a stable and uniform SEI film on the surface of the material. In addition, the observable decrease in the intensity of the reduction peak suggested the loss of capacity during the charge process. In the subsequent scan, the current peaks shifted to higher potentials due to the facile polarization on account of the good reversibility. Moreover, the shift of the oxidation peak to a high potential as the scan proceeds depends on the unique structure and high specific surface area of the composite materials, and is due to the reversible conversion reaction between Li^+^ and Fe_3_O_4_ simultaneously. The good overlap of the pattern in the subsequent scan verified the good reversibility of the composite.

(1) $$\mathrm{Sn}+x{\mathrm{Li}}^{+}+xe^{-}↔{Li}_{x}Sn\left(0<x<4.4\right)$$

(2) $${Fe}_{3}O_{4}+8{\mathrm{Li}}^{+}+8e^{-}↔4{Li}_{2}O+3\mathrm{Fe}$$

Figure 5c illustrates the corresponding discharge specific capacity of CNFs, Sn@CNFs, Fe_3_O_4_, Fe_3_O_4_@CNFs, and Fe_3_O_4_-Sn@CNFs at current densities of 100, 200, 400, 1600, and 100 mA·g^−1^. This test included fifty discharge and charge cycles and changed the current density every ten cycles. In the first forty cycles, the specific capacity constantly decayed with increasing current density because of the polarization phenomenon during the electrochemical reaction process. At the beginning of the 41st cycle, the applied current density was dropped back to 100 mA·g^−1^ to study the percent recovery of specific capacity by comparison to the initial ten cycles. Compared to the steady discharge capacity at various current densities and excellent percent recovery of the specific capacity of Fe_3_O_4_-Sn@CNFs, the Fe_3_O_4_ powder anode capacity quickly decayed from 720 to 410 mAh·g^−1^ at a high current density (1600 mA·g^−1^). After transforming the current density to 100 mA·g^−1^, the recovery ratio of the capacity of Fe_3_O_4_ powder reached 95%. The strongly stable discharge specific capacities of the Fe_3_O_4_-Sn@CNFs were 1120, 1030, 970, 785, and 1120 mAh·g^−1^ at current densities of 100, 200, 400, 1600, and 100 mA·g^−1^, respectively. The recovery ratio of the specific capacity was maintained at 99% during the last ten cycles, indicating the remarkable electrochemical stability of the materials which was closely related to the rate performance and electrochemical performance of the cell. The reason for the rate performance improvement was similar to that of the cycle performance, namely, the excellent cushioning effect and electrical conductivity of CNFs and the advantages of nanoscale Fe_3_O_4_ particles.

The cycle performance and coulombic efficiency of CNFs, Sn@CNFs, Fe_3_O_4_, Fe_3_O_4_@CNFs, and Fe_3_O_4_-Sn@CNFs at a current density of 100 mA·g^−1^ in the voltage range of 0.01–3 V are shown in Figure 5d. The initial discharge specific capacity of the prepared CNFs electrode was 464 mAh·g^−1^ and tended to be stable until the specific capacity fell to 208 mAh·g^−1^, with a coulombic efficiency of 99.4%, indicating that CNFs have excellent capacity retention properties and cycle performance despite the low capacity. Compered to pure CNFs, Sn@CNFs have a specific capacity with initial discharge specific capacity of 845 mAh·g^−1^ and are maintained at approximately 400 mAh·g^−1^ with a coulombic efficiency up to 99.5%, indicating that the loaded Sn effectively improved the electrochemical performance and specific capacity of the carbon fibers. Furthermore, the favorable cycling stability, remarkable specific capacity, and coulombic efficiency indicate that the CNFs commendably restrained the volume change of Sn during the intercalation and deintercalation process to a certain extent. The Fe_3_O_4_ powder electrode showed a high capacity of 1046.3 mAh·g^−1^ in the first discharge process, but the discharge/charge capacity decayed constantly until 582.5 mAh·g^−1^ at the 50th cycle of discharge capacity due to a large volume change in the cycle process. Compared to the initial discharge capacity of the Fe_3_O_4_@CNFs composites (1400 mAh·g^−1^), Fe_3_O_4_-Sn@CNFs have an amazing initial discharge capacity of 1600 mAh·g^−1^, and subsequently level off at approximately 1120 mAh·g^−1^ with little capacity fading and the coulombic efficiency stabilizes at approximately 98.5% simultaneously. These results suggest that Fe_3_O_4_-Sn@CNFs have excellent specific capacity and cycling stability. The electrochemical stability improvement of the Fe_3_O_4_-Sn@CNFs electrode was based on the following two primary factors: the doping of Sn (Sn@CNFs have excellent electrical conductivity) and the presence of Fe_3_O_4_ nanoparticles (nanoscale particles have higher electrochemical activity). Sn loading on CNFs effectively improved the electrochemical performance of the composite because of not only the high electrical conductivity of the materials but also the unique one-dimensional structure of the Sn@CNFs. Compared to carbon particles, one-dimensional fibers provide a 1D electron path which decreases the resistance during electron transfer and therefore expedites the lithiation and delithiation process. Fe_3_O_4_ nanoparticles grown on the surface of Sn@CNFs were separated from each other with high density, which means that more electrochemical reaction sites, larger specific surface area, and better electrolyte contact further improve the Li storage capacity. In addition, the active materials inevitably generate deformation during the lithiation process, especially Sn and Fe_3_O_4_ particles. With the lithium ion inserted into the composites, the nanofibers (with Sn and Fe_3_O_4_ integrated in the interior and exposed to the surface) expand and compress. At this point, CNFs play a crucial role to cushioning the mechanical stress caused by Fe_3_O_4_ and Sn because of their good elastic properties. Inhibiting the volume expansion of Fe_3_O_4_ and Sn can effectively restrain capacity fading.

The long-term cycling capacity at a high current density of 1600 mAh·g^−1^ was also measured to study the performance of the Fe_3_O_4_-Sn@CNFs anode in Figure 5e. The sample delivered first and second discharge capacities of 951 and 660 mAh·g^−1^, respectively, and retained the reversible capacity of 535 mAh·g^−1^ after 500 cycles, demonstrating its excellent electronic conductivity, good structural stability and capacity retention at a high discharge-charge current density.

**Figure 6:**
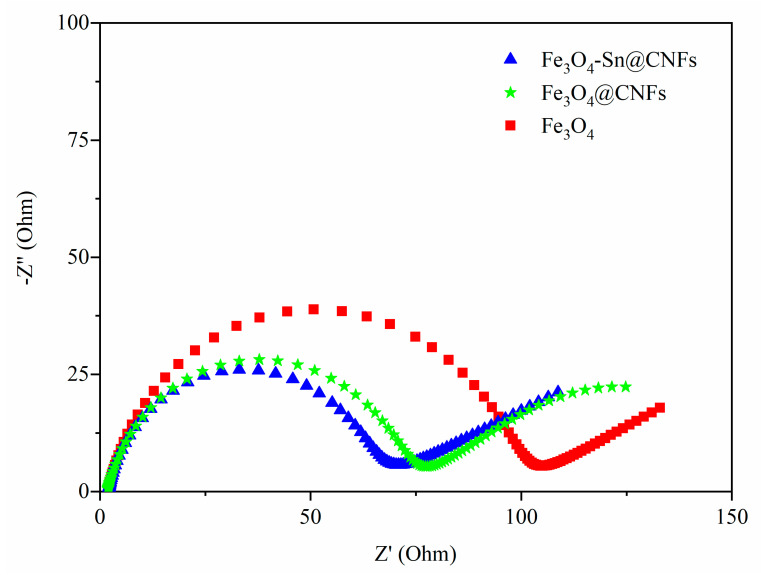
AC impedance spectra of Fe_3_O_4_, Fe_3_O_4_@CNFs, and Fe_3_O_4_-Sn@CNFs in 1M LiPF_6_/EC/DMC (EC: DMC = 1:1).

To further determine the kinetics of the as-prepared electrode materials during the charge-discharge process, electrochemical impedance spectroscopy (EIS) measurements were performed on Fe_3_O_4_ and Fe_3_O_4_@CNFs and Fe_3_O_4_-Sn@CNFs-based half cells. Figure 6 shows the Nyquist plot of the EIS curves with sinusoidal excitation signals in a frequency range of 100 kHz to 0.01 Hz. The EIS curves of anode materials for Li-ion batteries usually have a similar shape, including a semicircle in the high-frequency region and a straight line in the low frequency region. The intercept of the semicircle with real axis corresponds to the inherent resistance (Rs), including the active material resistance, electrolyte resistance, contact resistance between electrolyte and electrode, and contact resistance between electrode material and collector. The diameter of the semicircle represents the charge transfer resistance (R_CT_), which consists of a two-phase interface between the electrode and the electrolyte, reflecting the electron transfer ability at that interface and the difficulty of the electrochemical reaction. The curve of the oblique line in the low frequency region represents the Warburg impedance (Zw), corresponding to the lithium-diffusion process. The inherent resistances of Fe_3_O_4_, Fe_3_O_4_@CNFs, and Fe_3_O_4_-Sn@CNFs were almost the same (approximately 1.8 Ω), showing a high conductivity. A low R_CT_ is beneficial to electron transfer and enhances the electrochemical reaction kinetics of the materials. In this study, the R_CT_ of Fe_3_O_4_, Fe_3_O_4_@CNFs, and Fe_3_O_4_-Sn@CNFs were 90 Ω, 70 Ω, and 60 Ω, respectively. In comparison to the pure Fe_3_O_4_ powder, the R_ct_s of Fe_3_O_4_-Sn@CNFs was obviously much smaller, which provided the following benefits: (a) Sn@CNFs enhanced the conductivity of the active materials and the exchange of ions and electrons between particles via carbon fibers effectively promoting their transfer; (b) instead of large particles flocking together, the Fe_3_O_4_ that grew on the surface of the nanofibers was 8 nm, which observably reduced the internal resistance. Under the joint effect of the two above factors, the specific surface area of the active material increases, which facilitates the permeation of the electrolyte and charge transfer, decreases the charge transfer resistance, shortens the ion insertion distance in the nanometer range, and finally improves the electrochemical performance of the Fe_3_O_4_-Sn@CNFs anode.

## 4. Conclusions

Novel nanoscale Fe_3_O_4_-Sn@CNFs were prepared by loading Fe_3_O_4_ and Sn nanoparticles onto CNFs synthesized via electrostatic spinning and subsequent thermal treatment by solvothermal reaction and used as the anode material for lithium-ion batteries. The Fe_3_O_4_ nanoparticles play an important role in the improvement of the specific capacity, cycle stability, and rate performance of the electrode material. The initial discharge capacity of the Fe_3_O_4_-Sn@CNFs anode is 1600 mAh·g^−1^, which is much higher than that of the pure Fe_3_O_4_ powder (582 mAh·g^−1^), Fe_3_O_4_@CNF composites (900 mAh·g^−1^), and pure CNFs (200 mAh·g^−1^), and then falls to 1120 mAh·g^−1^ and stabilizes after fifty discharge/charge cycles without evident capacity decay. The rate performance of the Fe_3_O_4_-Sn@CNFs composites reveals excellent percent recovery of specific capacity (99%), which is far beyond that of the Fe_3_O_4_ powder. The excellent cycle stability and rate performance of the Fe_3_O_4_-Sn@CNFs anode benefit from the following factors: first, the low diameter and high density of nanoscale Fe_3_O_4_ particles at the surface of Sn@CNFs increase the electrochemical reaction sites and contact with the electrolyte, promote the transfer of lithium ions, obtain a faster velocity of lithiation, and improve the electrochemical performance. Second, Sn@CNFs have good electrical conductivity due to their unique one-dimensional structure, providing a 1D pathway of electrons, reducing the resistance, and tremendously facilitating the transfer of electrons during a lithiation and delithiation process. In addition, CNFs have commendable elastic properties, and generate cushioning effects to restrain the volume expansion, efficiently enhancing the rate performance and cycle capacity of the Fe_3_O_4_-Sn@CNFs composite material.

## Data Availability

The data presented in this study are available on a reasonable request from the corresponding author.
